# Sudden cardiac death and valvular pathology

**DOI:** 10.1080/20961790.2019.1595351

**Published:** 2019-08-19

**Authors:** Rosa H. A. M. Henriques de Gouveia, Francisco M. A. Corte Real Gonçalves

**Affiliations:** Forensic Clinical and Pathology Service, Central Branch of the National Institute of Legal Medicine and Forensic Sciences, Coimbra, Portugal

**Keywords:** Forensic sciences, forensic pathology, sudden death, cardiac valvular pathology

## Abstract

Sudden death due to valvular heart disease is reported to range from 1% to 5% in native valves and around 0.2%–0.9%/year in prosthesis. The nature of the diseases is varied, from heritable, congenital to acquired. It may affect both genders in multiple age groups. The authors show and comment examples of the major nosologic aetiologies underlying unexpected *exitus letalis* of valvular nature.

## Introduction

Sudden cardiac death (SCD) is defined as “a natural, unexpected fatal event, with a cardiac underlying cause, occurring instantly or within 1 h from the onset of symptoms in an apparently healthy subject or whose disease was not so severe as to predict an abrupt outcome” [1]. When not witnessed, it is considered sudden if “the deceased was in good health 24 h before death” [1]. Statistics on SCD vary with the cohorts/populations studied [2], age, gender, life-style and sports activity [1, 2]. Subjacent causes are multiple [3, 4] and valvular heart disease is reported to range from 1% to 5% [3]. After valve surgery, SCD takes place in 15%–30% of the patients, thus summing up to 0.2%–0.9%/year, mostly initiated by arrhythmias [3]. This article performs an overview of valvular sudden cardiac death (VSCD), by presenting cases of valve pathology leading to SCD.

## Case reports

The victims died sudden and unexpectedly in diverse settings. A complete postmortem examination was performed, including toxicological and anatomo-pathological evaluation. Toxicology was negative for alcohol, illicit drugs and pesticides. It was negative or in therapeutic doses for medicines. Organ samples were procured for microscopic examination. They were fixed in 10% formalin and embedded in paraffin. Microtome sections were stained with haematoxylin and eosin (HE). Additional special stains (Masson trichrome (MT) for fibrous tissue/collagen, Elastic van Gieson (EvG) for elastic fibres/tissue, Periodic Acid Schiff-Alcian Blue (PAS-AB) for mucins, *Gram* for bacteria) were performed on the heart samples, for better interpretation. Photography of the histological slides was done using Leica DM1000 LED microscope (Leica Microsystems, Wetzlar, Germany) and image acquisition system (Leica ICC50 HD) camera plus LAS EZ v2.0.0 for Windows software. The heart was macro- and microscopically studied by the Institute Anatomo-Pathologist (sub-specialized in Cardiovascular Pathology).

### Case 1

**Figure 1.**
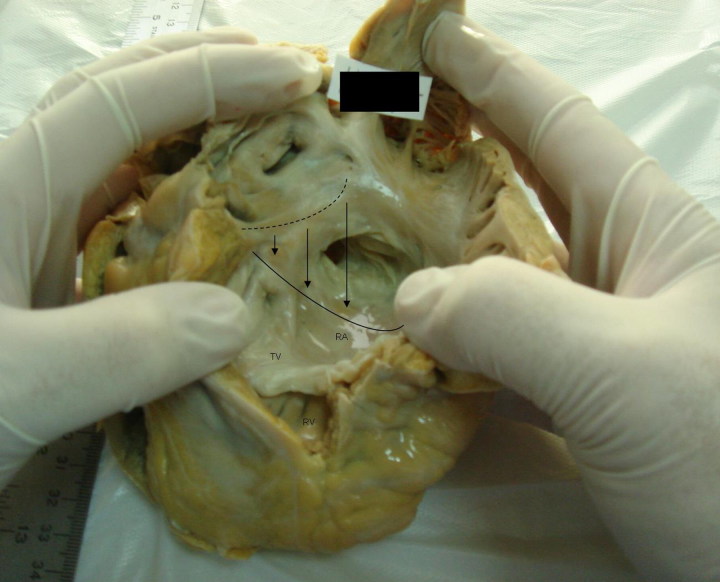
Macroscopic view of the right side of the heart, showing downward ventricular insertion of the tricuspid valve ring (“atrialization of the right ventricle”), leaflets partially adherent to the ventricular wall and free portion dysplastic. It also shows a right ventricle with minimal chamber and a “canal” outlet chamber to the pulmonary valve, due to the mal-positioned tricuspid leaflets (source: INMLCF, I.P., with permission).

A 44- year-old Caucasoid male, apparently healthy, apart from the occurrence of very rare “epileptic-like attacks”, died at work, while performing a minor effort task. His heart weighed 380.0 g and measured 14.7 cm × 12.0 cm, with global dilatation and congenitally malformed tricuspid valve, including downward ventricular insertion of the ring – “atrialization of the right ventricle” – as in the *Ebstein Disease* (Figure 1).

### Case 2

**Figure 2.**
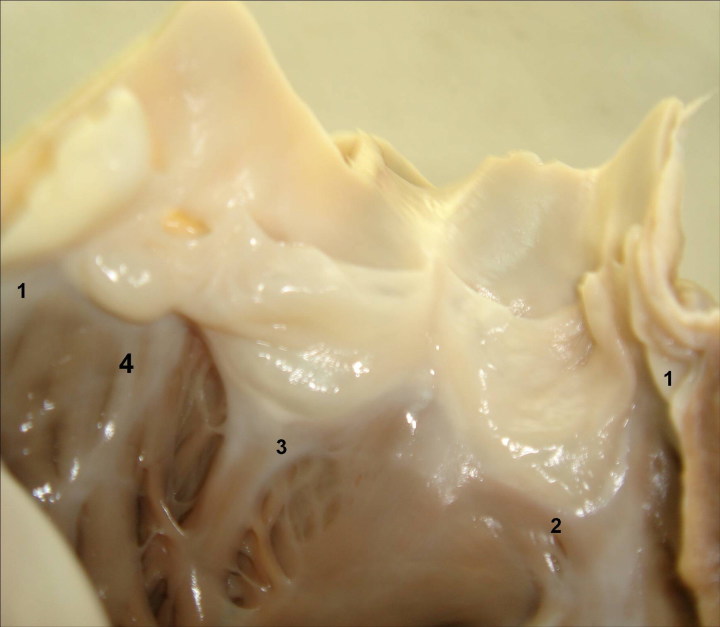
Macroscopic view of the Quadricuspid Pulmonary Valve, with three equal-sized cusps and an extra (No. 4) smaller one (source: INMLCF, I.P., with permission).

A 17-year-old Caucasoid male, with no relevant personal or familial pathologic antecedents or known risk factors, dropped dead at the platform, while waiting for the underground. His heart revealed nothing abnormal but a congenitally malformed *Quadricuspid Pulmonary Valve* (Figure 2).

### Case 3

**Figure 3.**
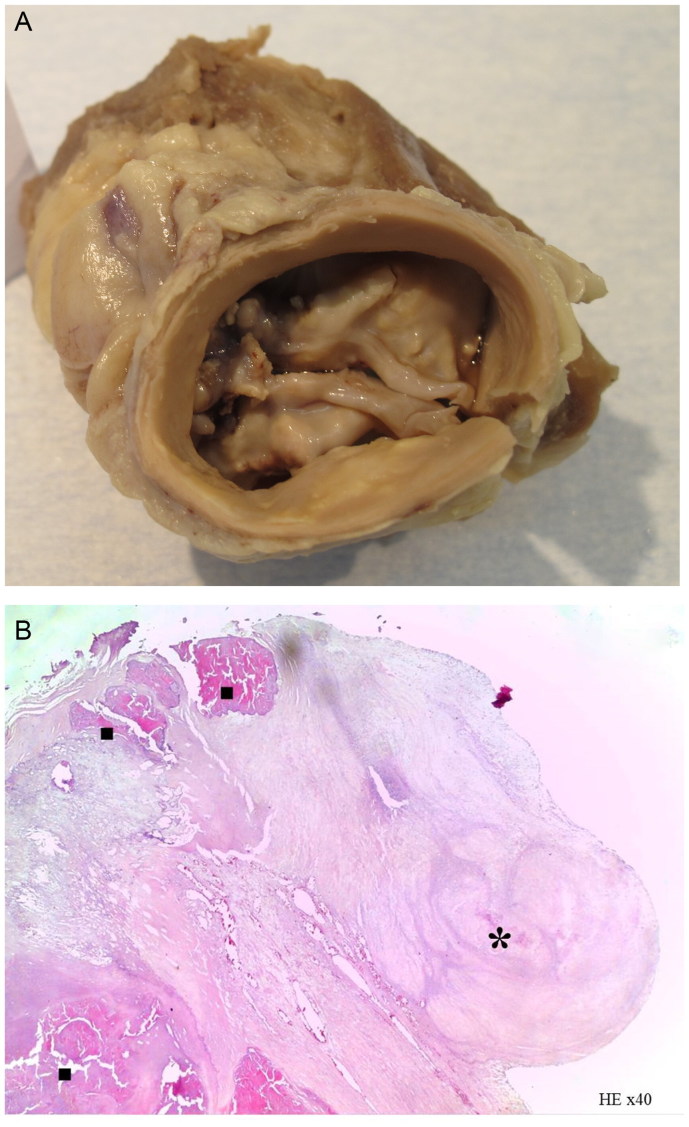
Macroscopic view of the Bicuspid Aortic Valve, type 1, showing major degenerative deformation (A), with fibro-sclerosing nodules (*) and dystrophic calcification (**■**) exhibited on the histological section (B: HE, ×40) (source: INMLCF, I.P., with permission).

A 48-year-old Caucasoid male, with no relevant personal or familial pathologic antecedents or known risk factors, was found dead at his house courtyard. His heart weighed 440.0 g, with hypertrophy and severe degenerative alterations of a congenitally malformed *Bicuspid Aortic Valve* (Figure 3).

### Case 4

**Figure 4.**
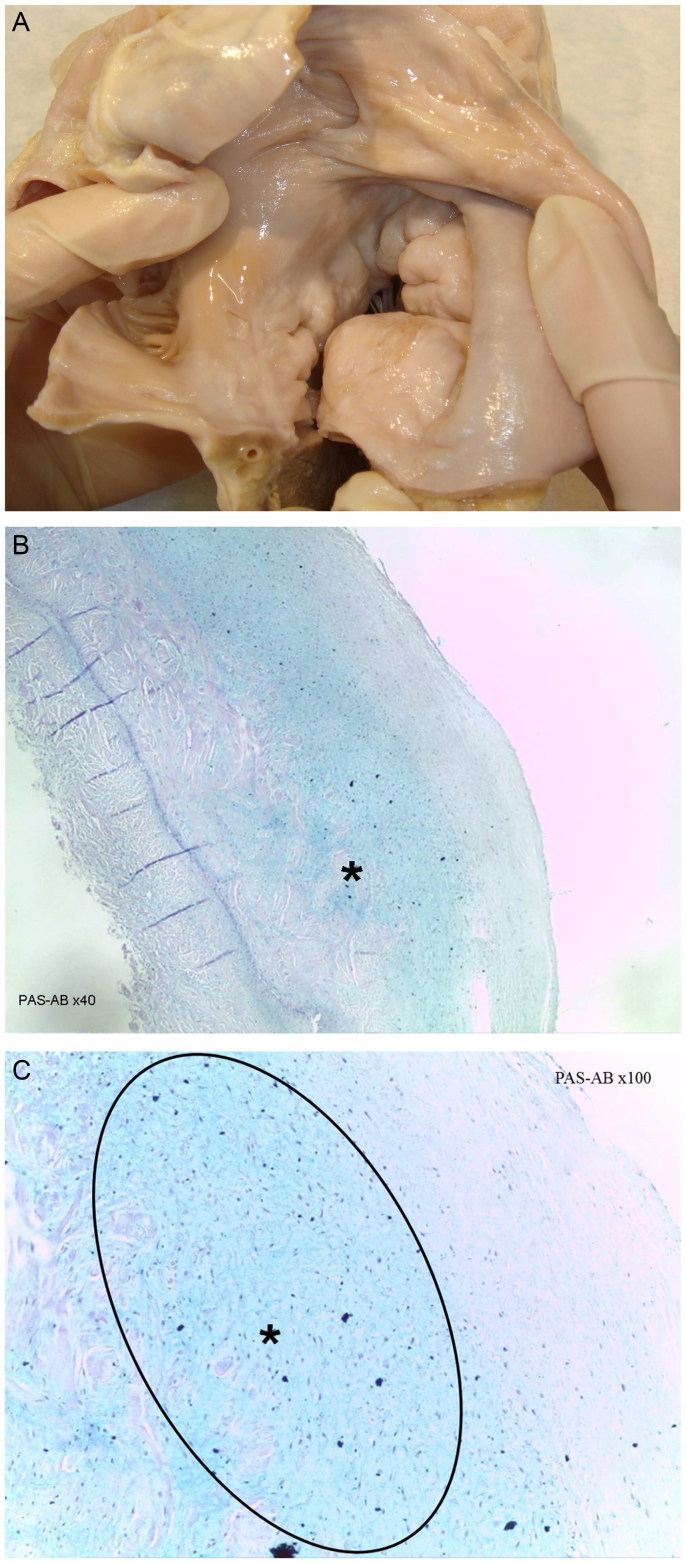
(A) Macroscopic view of the Floppy Mitral Valve, showing the upward ballooning and thickening of the leaflets. (B, C) Microscopic aspect of the mitral valvular acid mucoid deposits, which are Alcian Blue positive (* and oval area) (PAS-AB, ×40 and ×100) (source: INMLCF, I.P., with permission).

A 39-year-old Caucasoid female, with personal pathologic antecedents of syncope, died suddenly. Her heart weighed 362.5 g and measured 14.5 cm × 11.0 cm, with dilatation and prominent ballonization of the mitral valve leaflets, as in *Myxomatous Mitral Valve Prolapse* (Figure 4).

### Case 5

**Figure 5.**
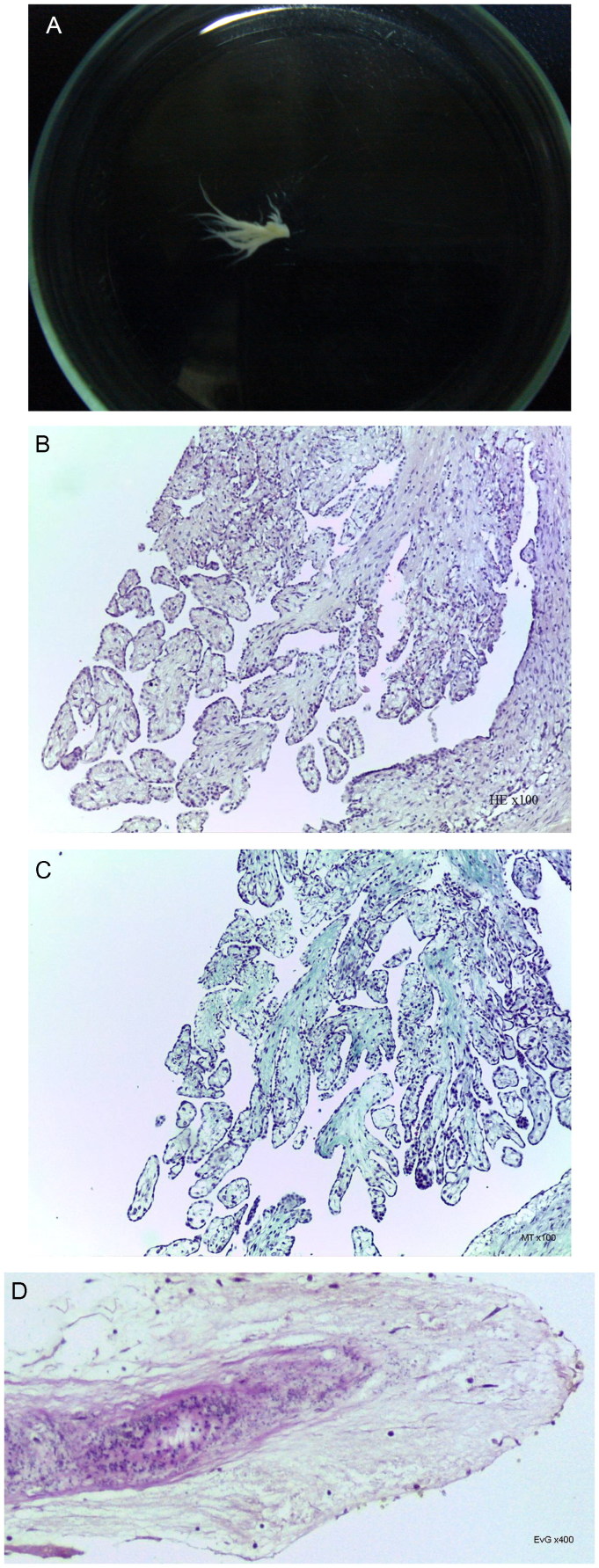
Macroscopic image (A). Histopathologic features of Cardiac Fibroelastoma, with papillary architecture (B: HE, ×100), fibrous axis of the stalk and branches (C: MT, ×100) and a closer view of the elastic component and the endothelial superficial monolayer (D: EvG, ×400) (source: INMLCF, I.P., with permission).

A 45-year-old Caucasoid male was found dead at home. The heart weighed 1 140.0 g, was dilated and presented a *Papillary Fibroelastoma* of the Pulmonary Valve (Figure 5).

### Case 6

**Figure 6.**
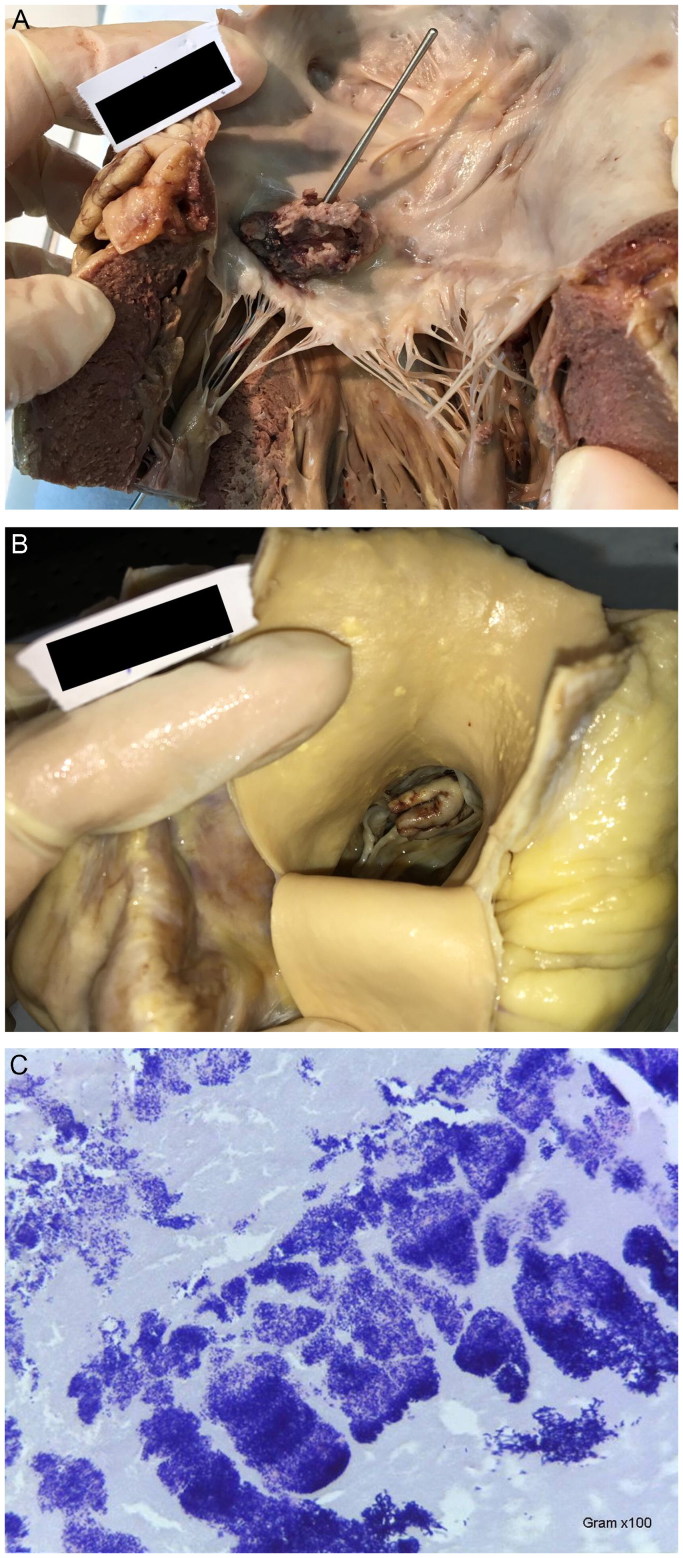
Macroscopic view of the vegetations (A) on the mitral valve, with leaflet perforation (B) on the aortic valve, with cusp tear. (C) Microscopic image of the Endocarditis vegetations with aggregates of Gram + bacteria (Gram, ×100) (source: INMLCF, I.P., with permission).

A 64-year-old Caucasoid male, with no relevant personal or familial pathologic antecedents or known risk factors, was found dead at home. The heart weighed 580.3 g, was hypertrophic and showed perforated, bi-valvular (mitral and aortic), *Infectious (Gram+ bacteria) Acute Endocarditis* (Figure 6).

### Case 7

**Figure 7.**
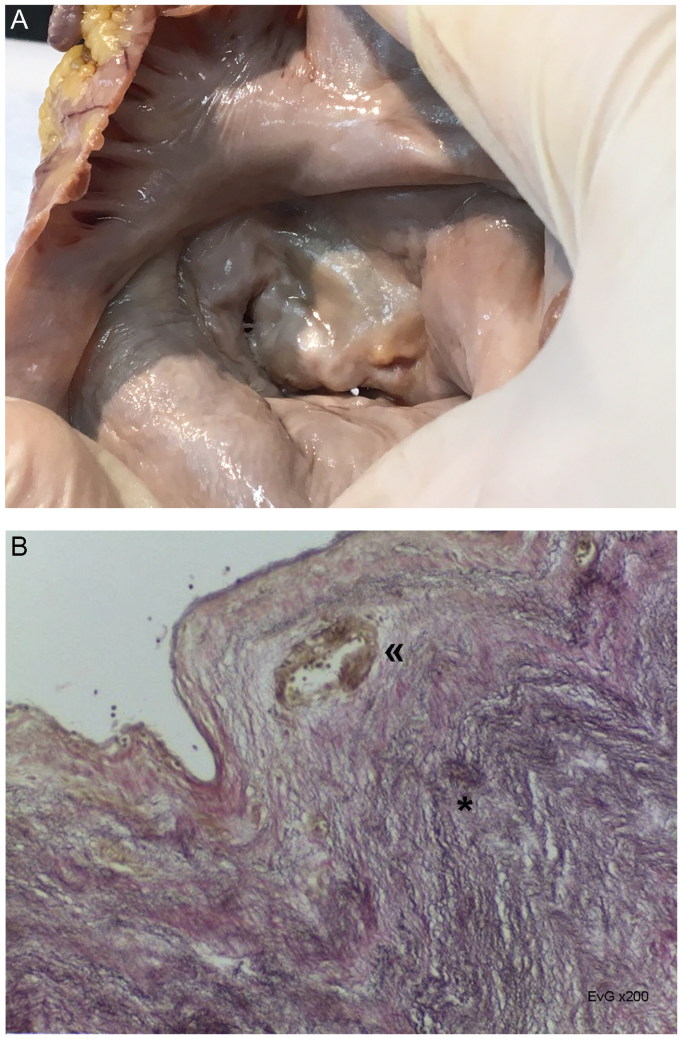
Macroscopic view of the mitral valve with thickening and commissures fusion, acquiring the aspect of “smiling face” or “fish mouth” (A). Microscopic image of Post-Inflammatory (Rheumatic) Valvulopathy, with fibroelastosis (*) and thick-wall vessels at the distal part of the leaflets (**«**) (B: EvG, ×200) (source: INMLCF, I.P., with permission).

A 70-year-old Caucasoid female, with personal medical history of Rheumatic Fever, was found fallen in the street. Cardiopulmonary resuscitation was performed while the victim was being transported to the hospital, where she died. The heart weighed 393.7 g, with hypertrophy and stenotic aortic-mitral valves, the latter displaying a “*smiling face*” or “*fish mouth*” pattern, suggesting *Post-inflammatory Valvular Cardiopathy*, of Rheumatic nature (Figure 7).

### Case 8

**Figure 8.**
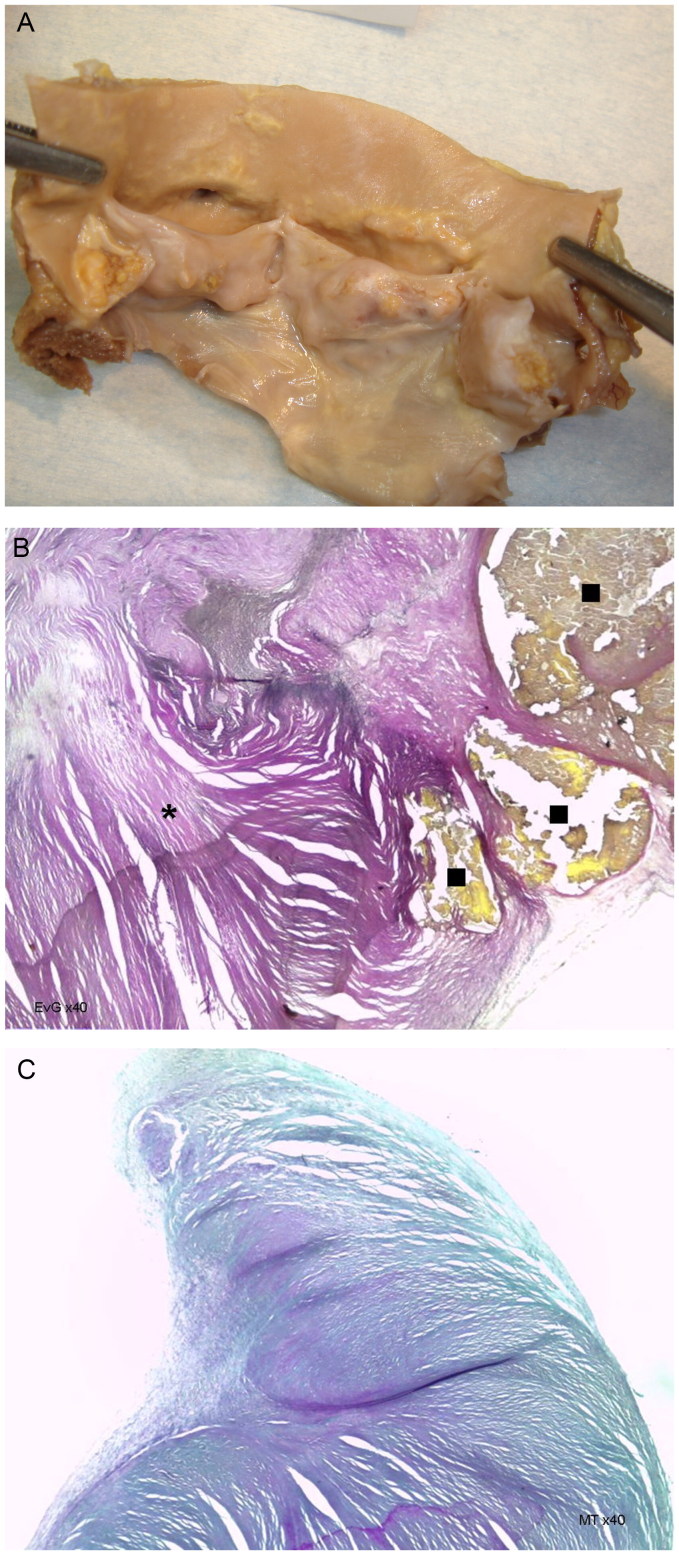
Macroscopic view of the Degenerative deformed aortic valve with thickened and extensively calcified cusps (A). Histopathologic features show calcifications (**■**), fibroelastosis (*) (B: EvG, ×40) and fibrosis of the cusps (C: MT, ×40) (source: INMLCF, I.P., with permission).

A 69-year-old Caucasoid male, with alcoholic and smoking habits and a previous stroke, was found at home, in advanced putrefaction state. The heart revealed severe mitro-aortic *Degenerative Valvular Cardiopathy* (Figure 8).

### Case 9

**Figure 9.**
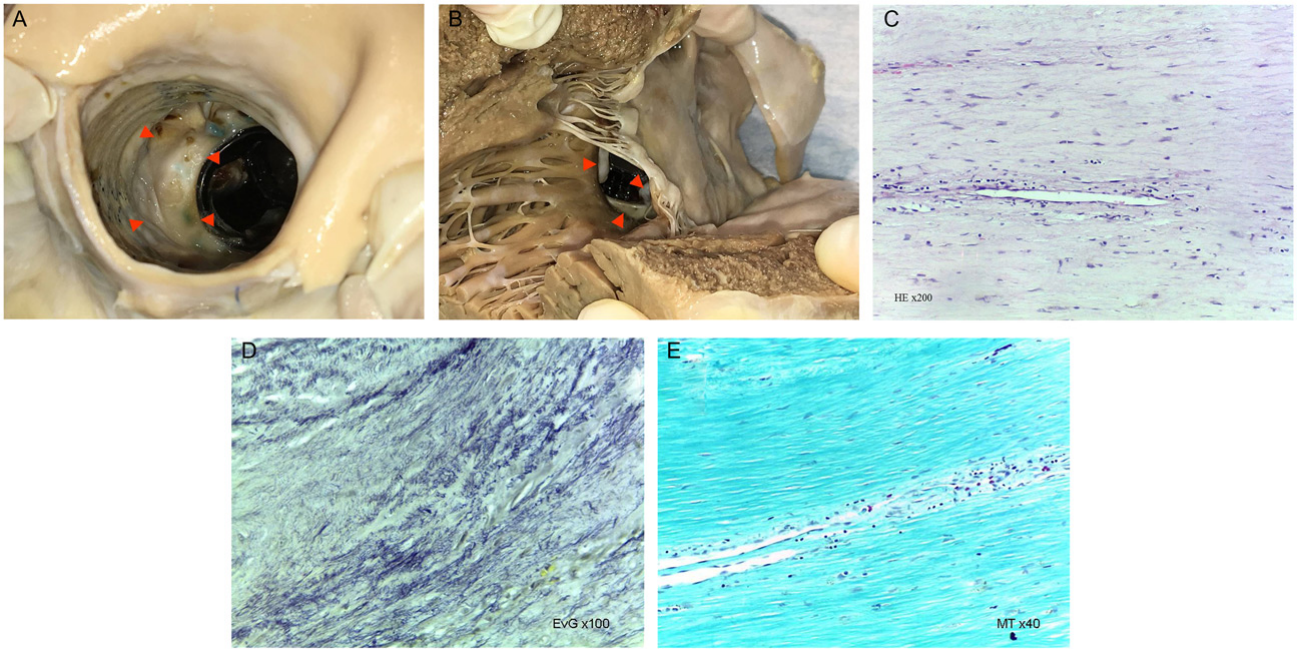
Macroscopic images of the *Pannus* (indicated by red arrowheads), partially covering the aortic valve prothesis (A: superior (aortic) view; B: inferior (left ventricular) view). Histopathologic features show rare capillary vessels, scarce mononuclear inflammatory cells (C: HE, ×200), fibroelastosis (D: EvG, ×100), and fibrosis (E: MT, ×40) (source: INMLCF, I.P., with permission).

A 72-year-old Caucasoid female, with a previous (5 years before) surgery, due to ascending aorta aneurysm, replacing the aorta by a synthetic conduit and the aortic valve by a bi-disc, metallic prothesis. She was found dead at home. The heart weighed 637.5 g, presenting ventricular hypertrophy and aortic valve prothesis closing and opening malfunction due to *Pannus* (Figure 9).

## Discussion

In the 21st century, Valvular Heart Disease may be isolated or affect multiple valves [5, 6]; and its aetiology may include 10 nosologic groups: heritable – congenital, inflammatory – immunologic, endocardial (infectious or not), myocardial or other organ diseases, neoplastic, degenerative, iatrogenic (even devices for valve repair or replacement), drugs and physical agents, infiltrative, and idiopathic [5, 7, 8]; all of which may underlie SCD, as showed by the cases reported.

*Ebstein Disease* – First described by Wilhelm Ebstein in 1866 [9], is a congenital anomaly due to failure of delamination of the tricuspid valve from the ventricular wall, leading to apical displacement of the valve leaflets into the right ventricle. It is esteemed to appear in 1 per 200 000 live births, mostly sporadic. It may be associated to other congenital malformations. Death may supervene to heart failure or arrhythmia. In fact, 3.5% of adult arrhythmic SCD in the setting of congenital cardiopathy is due to Ebstein Disease [9, 10].

*Quadricuspid Pulmonary Valve* – Is a rare congenital malformation (1 in 400–2 000 autopsies), with male predominance (2:1) [11, 12], resulting from one of the three valve cushions’ partition at a very early stage of valvulogenesis. Usually asymptomatic, may favour endocarditis, cardiac insufficiency and sudden death.

*Bicuspid Aortic Valve (BAV)* – Is a congenital malformation affecting 1%–2% of the population, with male predominance (3:1). It is the end product of abnormal valvulogenis, which differs with the cusps involved and has genetic background. The pattern of cusp fusion defines the type of BAV. It is associated to aortopathy. With age, degenerative alterations may aggravate the valve deformation, causing stenosis and/or insufficiency. Both the valvulopathy and the aortopathy may lead to unexpected sudden death [1, 13–17].

*Myxomatous Mitral Valve Prolapse* (*MVP*) – Also known as *Floppy Mitral Valve* or *Barlow’s Syndrome*, since it was first reported by Barlow in the 1968 [18]. Its prevalence ranges 2%–3%, with female and youth preference. It shows a fibroelastic deficiency with replacement by acid mucoid deposits, which stain bluish with Alcian Blue (AB) or PAS-AB. MVP presents a displacement of one or both mitral leaflets into the left atrium during systole. It was in 1984, that Pocock and co-workers [18] refered to the association between Barlow’s Disease and Sudden Death, which is esteemed in 0.46–3.7 per 100 000 persons-years and may occur due to myocardial dysfunction, embolism, endocarditis, autonomic dysfunction/syncope and arrhythmias [18, 19].

*Papillary Fibroelastoma* – Named by Gowda et al. in 2003 [20], is the most frequent valvular begnin tumour. It may be seen in every valve, from the aortic (35%–63%) to the pulmonary (0.5%–8%). Its frailty and mobility may lead to fatal or non-fatal embolization [20].

*Infectious Endocarditis* – Is esteemed to occur in 3–10 persons per 100 000 per year. Any age group may be affected, depending on the virulence of the microorganisms and on the risk factors of the victim, namely the presence of a congenital malformation. There is out-of-hospital forms and nosocomial ones. It may involve one or more valves, native and/or prosthetic; and it may extend to other cardiac areas/tissues or systemically through septic embolization. Sudden Death has been reported to range 2.7% [21–23].

*Post-inflammatory Valvular Cardiopathy* – Is an inflammatory/immunologic-based group of valvular lesions, mostly in the setting of a systemic disease. An example is the Rheumatic Heart Disease – the result of previous Acute Rheumatic Fever, still an important cause of valve pathology (15 million persons affected worldwide), although underestimated. The involvement may be uni- or multivalvular (usually mitral and aortic valves), with stenosis, regurgitation or both. Suprajacent pathology, like endocarditis, thrombosis, is frequent pathology [24, 25].

*Degenerative Valvular Cardiopathy* – Is the most frequent valvular pathology of the present time. It is due to environmental and genetic predisposing factors, often acting together. It is favoured by the rise of life expectancy, with tissue senescence; despite also appearing upon a congenital malformation or a previous valvular/heart lesion. Fibrosis, fibroelastosis, deposits of anomalous substances, atherosclerosis, calcification are some of the morphologic features, and may involve any component of the atrioventricular or semilunar valve apparatus, as for example calcification of the aortic cups (as in the case here presented) or of the mitral annulus (as reported in autopsy series around 8.5%) [26–29].

*Pannus* – Is one of the complications of prosthetic heart valves. It is not acute and corresponds to tissue overgrowth from the edges/annulus, eventually causing opening and/or closing dysfunction of the valve leaflets/cusps. The incidence is esteemed in 1.6%–2%, with female predominance [30, 31].

## Conclusion

The examples presented in this article emphasize the contribution of valvular pathology to sudden and unexpected *exitus letalis* of cardiac nature, in young, adult and old persons.
